# Variant-specific RNA testing resolves variants of uncertain significance in exome testing

**DOI:** 10.1186/s12864-026-13094-y

**Published:** 2026-07-01

**Authors:** Audrey K. O’Neill, Grace E. VanNoy, Brooklynn Gasser, Jessica Gage, Jessica Grzybowski, Carolyn Horton, Melissa Holman, Claire Watson, Shoji Ichikawa, Changrui Xiao, Dana Mittag, Edwin C. Ferren, Erika Levine, Mafalda Barbosa, Joseph Biddle, Laurie Sadler, Melissa Samons, Heather Zimmermann, Marcy E. Richardson

**Affiliations:** 1 Ambry Genetics, 1 Enterprise Drive, Aliso Viejo, California 92656 USA; 2 Department of Neurology, University of California Irvine Health, Orange, CA USA; 3 Levine Children’s Hospital, Atrium Health, Charlotte, NC USA; 4 Division of Medical Genetics and Genomics, Department of Genetics and Genomic Sciences, Icahn School of Medicine at Mount Sinai, New York, NY USA; 5 The Mindich Child Health & Development Institute, Icahn School of Medicine at Mount Sinai, NY New York, USA; 6 Division of Genetics, Department of Pediatrics, Jacobs School of Medicine and Biomedical Sciences, University at Buffalo, Buffalo, NY USA

**Keywords:** RNA analysis, Variants of uncertain significance, Rare disease

## Abstract

**Background:**

Variants of uncertain significance (VUS) are a substantial barrier to clinical care in genetics, and VUS that are suspected to affect splicing can be particularly difficult to resolve. We used exome sequencing (ES) followed by variant-specific RNA testing to resolve VUS in four separate cases resulting in reclassification to Likely Pathogenic and a positive molecular diagnosis in all cases.

**Results:**

The variants in these cases include a homozygous variant in *ATP6V0A2* in a trio exome case, heterozygous variants in *SLC20A2* and in *CSF1R* in adult proband-only cases, and a heterozygous variant in *COL1A1* in a family. All variants were associated with aberrant splicing resulting in loss of function or a major alteration to the protein sequence.

**Conclusions:**

These findings demonstrate the power of targeted RNA testing for reclassifying VUS that have limited direct evidence across a broad range of indications for exome testing.

## Background

Exome sequencing (ES) is now a first-line tool for a variety of indications; however, 20–25% of patients tested on exome still receive a variant of uncertain significance (VUS) [1] following genetic testing. The resolution of VUS is a major focus in the field of clinical genetics [2]. Intronic and synonymous variants that are suspected to alter splicing in a rare disease gene can be particularly difficult to classify [3]. Variants at the canonical splice site (i.e., altering a nucleotide at +/- 1 or 2) are often eligible for strong pro-pathogenic evidence based on a predicted splicing impact, assuming they meet certain requirements [4, 5]. However, the impact of variants that are outside the canonical splice site is harder to predict, necessitating experimental evidence of a splicing impact to apply weight based on the nature and magnitude of the splice effect. While splice prediction tools can be useful for prioritizing the reporting of intronic and synonymous variants of interest, in the absence of sufficient other data they do not provide enough evidence to classify a variant as pathogenic or benign [6]. In addition, although these tools can make predictions about whether a splice impact is likely to occur, false positives are possible [7] and there is inherent uncertainty about the magnitude of impact and the ultimate effect on the protein. This makes the interpretation of a splice effect based on these tools alone risky. Variants that alter splicing can have one or more impacts, including full or partial exon skipping, inclusion of an intronic cryptic exon, partial intron inclusion resulting in extension of an exon, and full intron retention [8, 9]. Any of these types of events could potentially be either benign or pathogenic. Therefore, variant classification schemes should be conservative when applying weight for splicing predictors, especially when the variant is outside of +/- 1,2 position and/or when multiple predicted effects are likely.

When a variant suspected to impact splicing is reported as a VUS after ES, variant-specific RNA analysis can be a powerful tool to reclassify the variant and provide a diagnosis. This approach has shown promise in a variety of applications. In a US-based cohort, approximately 15% of cases without a conclusive diagnosis on ES or genome sequencing received a molecular diagnosis after RNA follow-up testing [8]. In a primary immunodeficiency study, RNA data provided supportive evidence towards pathogenicity in over half the patient cohort; notably, all the variants in this cohort were exonic, showing that the utility of RNA testing is not limited to intronic variants [6]. In addition, because RNA-based evidence does not rely on population data, it can be useful for reclassifying variants seen in understudied populations [10] (although the structural barriers to accessing ES and follow-up testing for these populations remain). At our laboratory, careful patient selection based on phenotype, family history, and presence of a potentially spliceogenic VUS, followed by targeted RNA analysis, has been useful for resolving VUS. To identify the variants that would benefit the most from RNA testing, this laboratory uses the following criteria: (1) the variant is suspected to be spliceogenic based on variant position and/or splicing predictions; (2) the variant is in a gene that is expressed in the available sample type (namely blood); (3) the variant is in a characterized gene with at least moderate gene-disease validity (GDV) per our laboratory’s internal GDV curation [11] ; and (4) the variant is in a gene for which the mechanism of disease is consistent with a splicing impact (usually loss of function (LOF)). In addition, the associated disease must have reasonable phenotypic overlap with the proband’s indication for exome testing and the variant zygosity must match the disease’s mode of inheritance. Overall, around 7% of all variants reported on exome, including 67% of suspected spliceogenic variants, meet these four criteria [12], showing that this approach is broadly applicable.

There is an urgent need for molecular diagnoses in rare disease, as this can facilitate tailored clinical care, trial eligibility, familial cascade testing, family planning, and other possible beneficial outcomes. Given this need and the high VUS rate in ES, we have developed a Reverse Transcription Polymerase Chain Reaction sequencing (RT-PCRseq) approach for targeted RNA testing in cases where a variant seen on ES could be reclassified based on RNA evidence. In this study, we describe four such variants and illustrate the broad applicability of RNA testing across disease areas, inheritance patterns, and disease mechanisms.

## Results

We present four cases in which variants detected by exome sequencing were reported based on strong clinical overlap and appropriate zygosity for the disease of interest (Table 1). However, because of limited clinical and functional evidence for these variants, they were initially reported as VUS. All of the variants were eligible for RNA testing based on our internal criteria as described in the Introduction. Upon follow-up RT-PCRseq, we detected a substantial increase in aberrant splicing (expressed as Percent Splicing Index (PSI), which is a measure of the relative number of reads supporting a splicing event), in all four cases compared to controls (*n* = 3 controls per case; see Methods). In each case, the additional functional evidence provided by follow-up RT-PCRseq allowed the VUS to be reclassified as likely pathogenic, providing a molecular diagnosis for the proband (and, in Case 4, for their affected relatives).

**Table 1:** Clinical and genetic findings in our cases

Case	Testing	Zygosity	Gene	Variant	Clinical summary	Associated splice event
1	Trio ES	Hom	*ATP6V0A2* MIM: 611716 HGNC: 18481	c.2055+A>C	Dysmorphic features; global developmental delay; hypotonia; febrile seizure; poor vision; tenting of skin; lissencephaly; sensory integration dysfunction	Skipping of coding exon 16: r.1936_2055del (p.E646_R685del); ~99% PSI
2	Singleton ES	Het	*SLC20A2* MIM: 158378 HGNC: 10947	c.935-3 C>G	Brain MRI: calcifications in the basal ganglia and dentate gyrus; psychosis with paranoid delusions; cognitive impairment	Skipping of coding exon 7: r.935_1523del (p.G312Vfs*8); ~52% PSI
3	Singleton ES	Het	*CSF1R* MIM: 164770 HGNC: 2433	c.2442+5G>A	Brain MRI: progressive white matter changes and cerebral volume loss; cognitive changes; ataxia; seizures	Skipping of coding exon 17 with or without an accompanying insertion (see text and Fig. 3); ~59% PSI
4a	Duo ES	Het	*COL1A1* MIM: 120150 HGNC: 2197	c.859-17_859-16delinsGTAGATCAGAAT	Prenatal ultrasound at 2 months gestation: small chest; leg length discrepancy; short femurs; bowed legs	Inclusion of part of intron 13: r.858_859insAATTTCTCTGATCTACAG (p.K286_G287insNFSDLG); ~18% PSI
4b	Duo ES (parent of 4a)	Het			Short stature; short long bones; low impact fractures; wormian bones; varus bowing of the proximal-mid femoral diaphysis	
4c	Previous testing* (sibling of 4a)	Het			Short stature; short long bones; low impact fractures; wormian bones; varus bowing of the proximal-mid femoral diaphysis	

*MIM* Online Mendelian Inheritance in Man gene identifier, *HGNC* HUGO Gene Nomenclature Committee gene identifier

*The sibling in family 4 was previously tested at an outside laboratory and found to carry the *COL1A1* variant

### Case 1

**Fig. 1:**
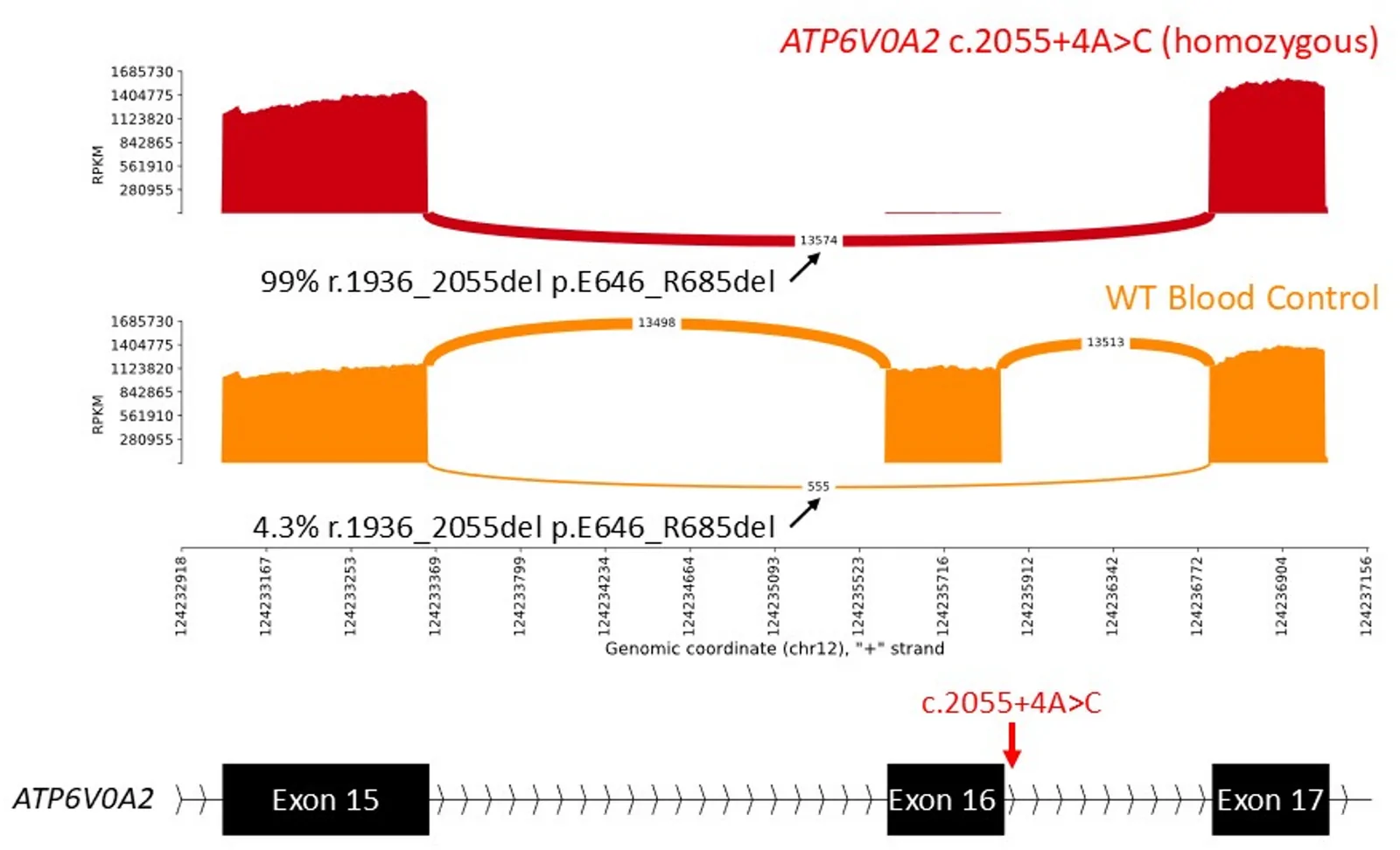
RT-PCRseq results for Case 1 (*ATP6V0A2* c.2055+4A>C). Top: plot depicting near-complete skipping of coding exon 16 in the proband (red) but not in a representative wildtype (WT) control (orange). Two other WT controls had low levels of splicing similar to that of the WT control depicted here (3-5%; data not shown). Bottom: the intron-exon structure is depicted to scale with chromosomal locations (hg19), and the location of the variant in red text. The number of reads supporting each splice event is provided on the connecting arched line. Splice events occurring at less than 2% PSI are filtered out.

Case 1 was a young child with tented, hyperextensible skin, including wrinkling of the hands and around the neck, dysmorphic facial features, microcephaly, global developmental delay, and one febrile seizure in early childhood, among other features consistent with *ATP6V0A2*-related cutis laxa. This proband had undergone multiple rounds of panel and exome testing at outside laboratories without a diagnostic result. Notably, previous ES at an outside laboratory had revealed the presence of a homozygous intronic *ATP6V0A2* variant, NM_012463.3:c.2055+A>C. This variant was confirmed to be homozygous in the proband and heterozygous in the unaffected parents on trio ES at our laboratory. SpliceAI strongly predicted loss of the native donor splice site with a donor loss score of 0.53. Due to a lack of other clinical or functional evidence, the variant was initially classified and reported as VUS. Subsequent RT-PCRseq of the relevant region revealed near-complete skipping of coding exon 16 in the proband sample compared to controls (PSI ~ 99% vs. 3–5%) (Fig. 1). This splicing event results in r.1936_2055del, which is predicted to result in an in-frame impact (p.E646_R685del) affecting 4.6% of the protein. Deletion of exon 16 has been reported as homozygous in multiple probands with cutis laxa [13, 14], providing support for the clinical relevance of the deleted region. Based on this finding, we applied additional RNA functional evidence, allowing us to reclassify the variant as Likely Pathogenic.

### Case 2

**Fig. 2:**
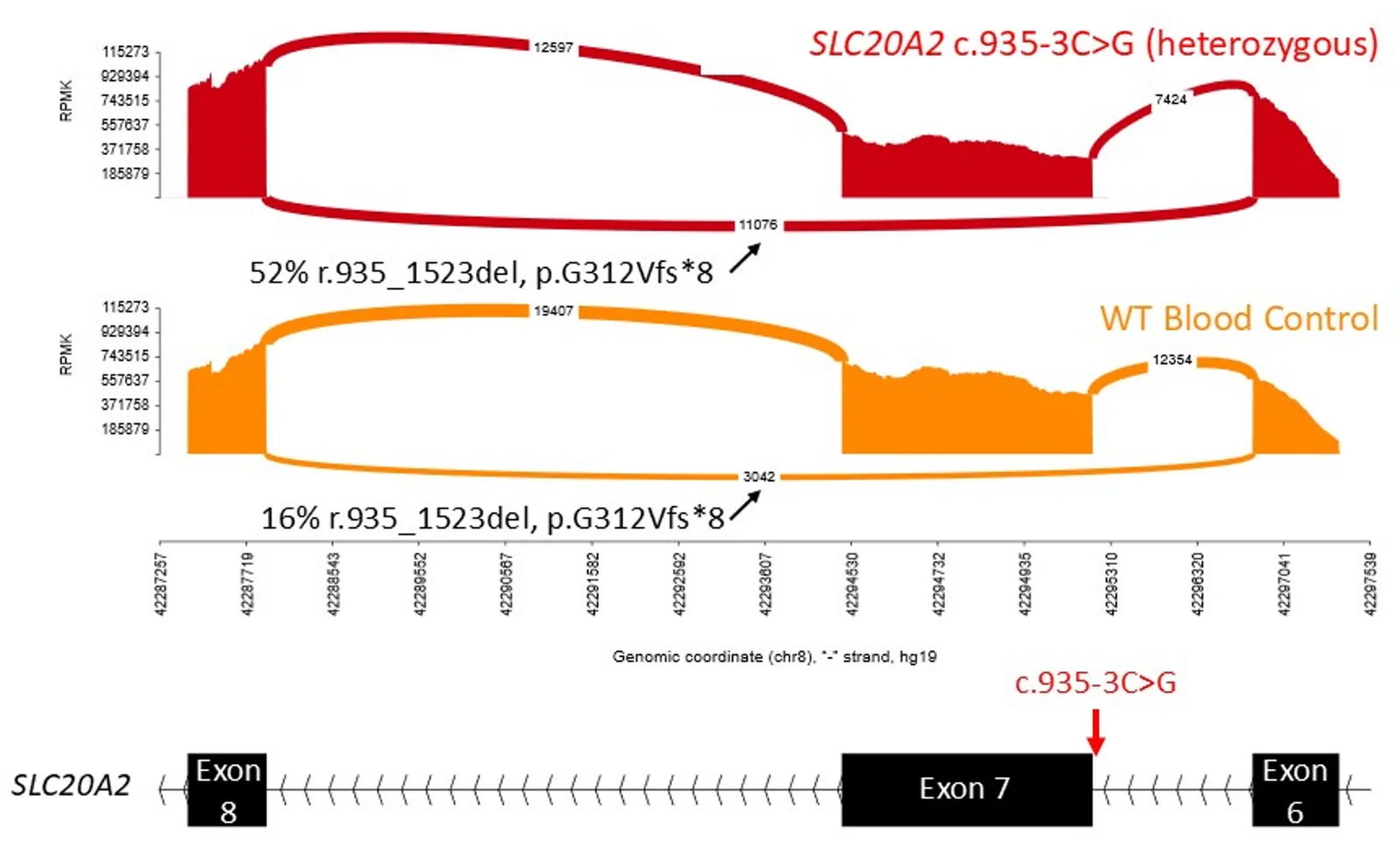
RT-PCRseq results for Case 2 (*SLC20A2* c.935-3C>G). Top: plot depicting skipping of coding exon 7 in the proband (red) at increased levels in the proband compared to a representative wildtype (WT) control (orange). Two other WT controls had low levels of splicing similar to that of the WT control depicted here (16-29%; data not shown). Bottom: the intron-exon structure is depicted to scale with chromosomal locations (hg19), and the location of the variant in red text. The number of reads supporting each splice event is provided on the connecting arched line. Splice events occurring at less than 2% PSI are filtered out.

Case 2 had brain imaging (magnetic resonance imaging and computed tomography) showing ectopic calcifications in the basal ganglia and dentate gyrus concerning for Fahr’s syndrome (familial primary brain calcifications). Symptoms included psychosis with paranoid delusions and cognitive impairment, with symptom onset in middle age. Previous panel testing at an outside laboratory had revealed a novel heterozygous variant in *SLC20A2*, NM_006749.3:c.935-3C>G, which was confirmed on proband-only ES at our laboratory (variant previously reported [12]). SpliceAI predicted loss of the native acceptor splice site with an acceptor loss score of 0.54. There was no known family history of disease, and other family members were not available for imaging or testing; therefore, in the absence of other clinical or functional evidence, the variant was initially classified and reported as VUS. Subsequent RT-PCRseq of the relevant region revealed increased skipping of coding exon 7 in the proband sample compared to three controls (PSI ~50% vs 16-29%) (Fig. 2). This splicing event results in r.935_1523del, which is predicted to result in a nonsense-mediated decay-prone frameshift (p.G312Vfs*8), consistent with the known loss-of-function mechanism for *SLC20A2*-related primary brain calcifications. Although we are unable to confirm statistical significance given the small sample size, the magnitude of the splice impact in our proband (~50% PSI, Fig. 2) was larger than that in our tested controls (16-19% PSI) and the splice impact is congruous with the skipping of coding exon 7 reported for another variant (NM_006749.3:c.935-1G>A) impacting the same splice site, which was detected in a proband and parent with clinical and imaging features similar to those of our case [15]. We therefore applied additional RNA functional evidence and reclassified the variant as Likely Pathogenic.

### Case 3

**Fig. 3:**
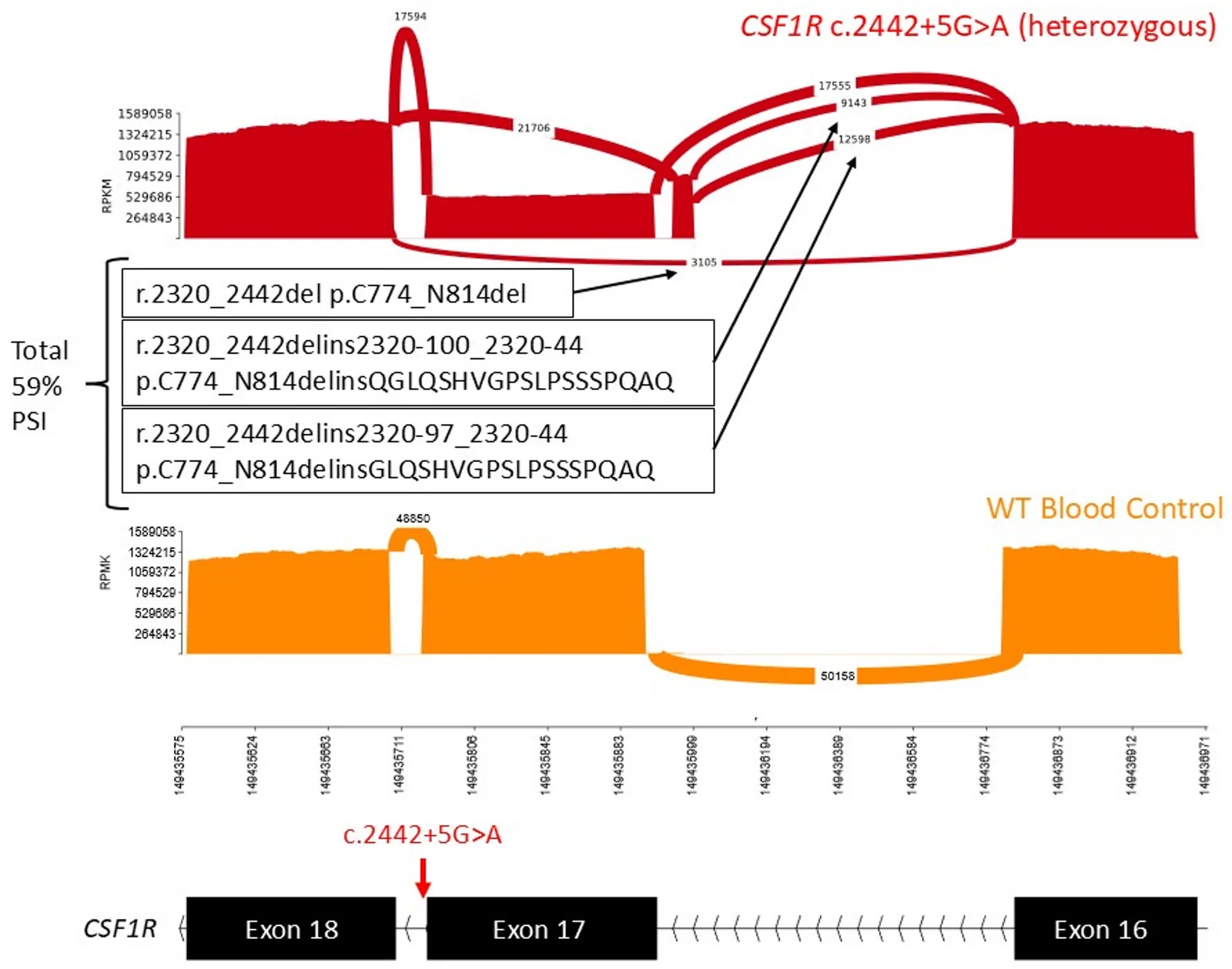
RT-PCRseq results for Case 3 (*CSF1R* c.2442+5G>A). Top: plot depicting skipping of coding exon 17 (r.2320_2442del), sometimes in combination with inclusion of a cryptic exon (r.2320_2442delins2320-100_2320-44 or r.2320_2442delins2320-97_2320-44) in the proband (red) but not in a representative wildtype (WT) control (orange). The total PSI for these aberrant transcripts was ~59% in the proband sample. Two other WT controls lacked these aberrant splice events, as in the WT control depicted here (data not shown). Bottom: the intron-exon structure is depicted to scale with chromosomal locations (hg19), and the location of the variant in red text. The number of reads supporting each splice event is provided on the connecting arched line. Splice events occurring at less than 2% PSI are filtered out.

Case 3 was suspected to have a genetic form of adult-onset leukodystrophy based on brain MRI findings showing progressive white matter changes and cerebral volume loss, as well as cognitive changes, ataxia, and seizures with onset in the early 50s. Previous panel testing at an outside laboratory had revealed a heterozygous variant in *CSF1R*, NM_005211.3:c.2442+5G>A, along with several other non-diagnostic variants. Notably, this variant has been previously reported as present in a large adult-onset leukoencephalopathy cohort [16]. The *CSF1R* variant was confirmed and reported on proband-only ES at our laboratory. While this variant was not predicted to impact splicing by SpliceAI (scores for donor loss and donor gain were both 0.01), it is located within the consensus donor site sequence of coding intron 17. Multiple other variants located within this donor site have also been reported in individuals with autosomal dominant *CSF1R*-related diffuse leukoencephalopathy with spheroids. Several of these variants have published RNA evidence showing in-frame skipping of coding exon 17 (often referred to as exon 18 in the literature) in combination with cryptic exon inclusions of 54 or 57 base pairs [17–21]. These aberrant transcripts are predicted to result in three in-frame protein impacts, p.C774_N814del, p.C774_N814delinsGLQSHVGPSLPSSSPQAQ and p.C774_N814delinsQGLQSHVGPSLPSSSPQAQ, respectively. Therefore, although the mechanism of disease for autosomal dominant *CSF1R*-related leukoencephalopathy is not loss-of-function, the likely splicing effect for our variant of interest (c.2442+5G>A) was consistent with the known variant spectrum for this disease. However, given the lack of a splicing prediction and the non-specific phenotypic findings, the variant was initially classified as VUS. Subsequent RT-PCRseq of the surrounding region revealed several aberrant splicing events present in our proband and absent in controls, all of which included the predicted exon skipping with or without in-frame cryptic exon inclusion events (Fig. 3). Our results demonstrated a splicing impact similar to those previously published in RNA studies for other variants disrupting this donor site, with a total PSI for aberrant transcripts exceeding 50%. Based on this RNA finding and the known association between deletion of coding exon 17 and the disease phenotype, we applied additional RNA functional evidence, resulting in reclassification of the variant to Likely Pathogenic.

### Case 4

**Fig. 4:**
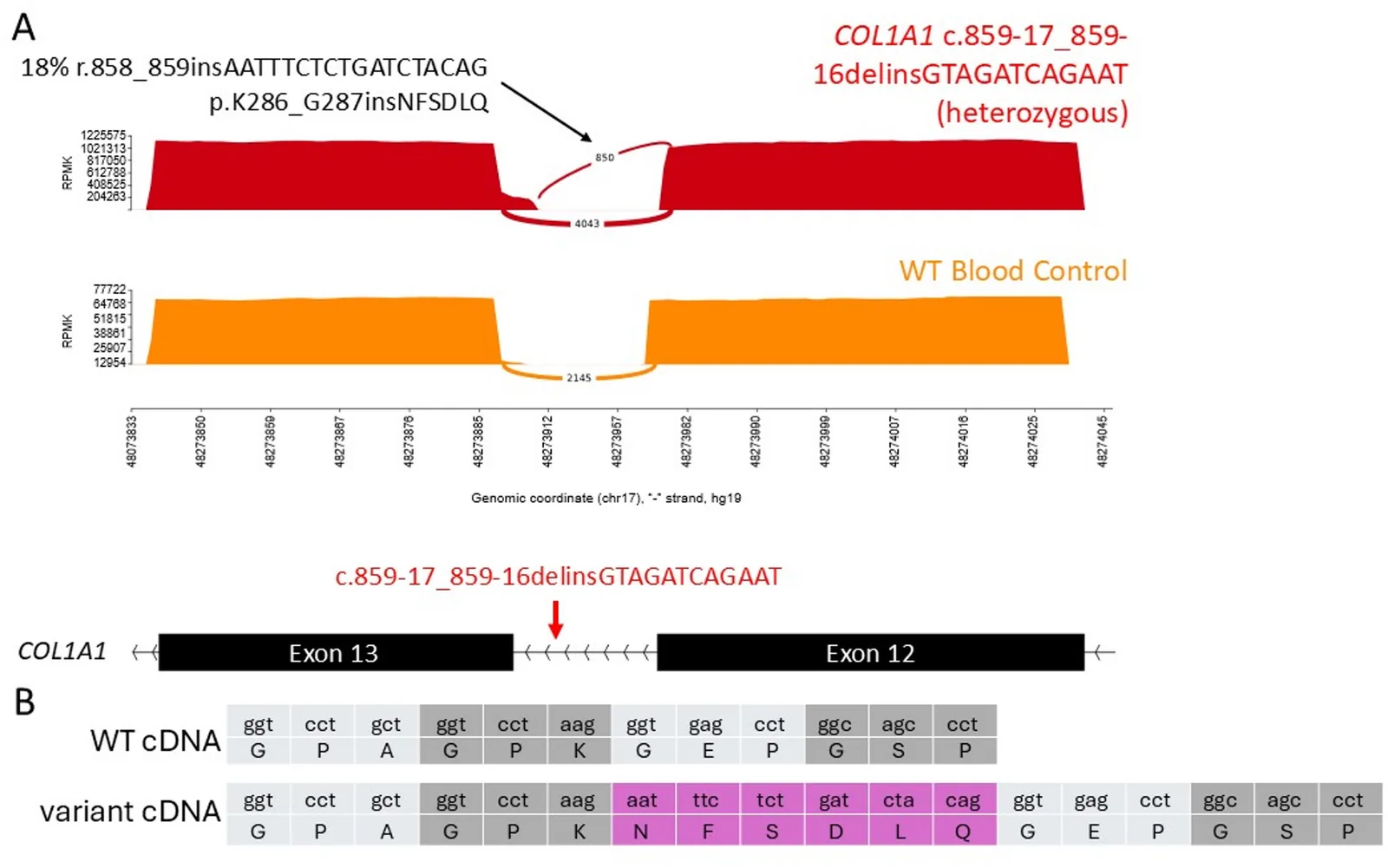
A) RT-PCRseq results for Case 4 (*COL1A1* c.859-17_859-16delinsGTAGATCAGAAT). Top: plot depicting inclusion of a portion of intron 13 (r.858_859insAATTTCTCTGATCTACAG) in the proband (red) but not in a representative wildtype (WT) control (orange). Two other WT controls lacked these aberrant splice events, as in the WT control depicted here (data not shown). Bottom: the intron-exon structure is depicted to scale with chromosomal locations (hg19), and the location of the variant in red text. The number of reads supporting each splice event is provided on the connecting arched line. Splice events occurring at less than 2% PSI are filtered out. B) The 18 bp cDNA insertion in the proband is predicted to disrupt the triple helical domain of *COL1A1* (p.179-1187) by introducing a non-triplet sequence (NFSDLG, in purple) between two G-X-Y repeats (in gray).

The proband in case 4 was diagnosed with autosomal dominant osteogenesis imperfecta (OI) type 1 prenatally based on family history and ultrasound findings including leg length discrepancy, short femurs, and bowed legs. Upon postnatal examination at 2 months of age, grey sclera was noted, but there were no fractures and no bowing or deformities of the limbs. The family history was notable for clinical features consistent with OI (notably grey sclera, short stature, short long bones, low impact fractures, and X-rays showing Wormian bones) in a parent and older sibling. The parent and older sibling were both known to carry a *COL1A1* VUS, namely NM_000088.3:c.859-17_859-16delinsGTAGATCAGAAT. This variant was detected on duo ES (for the proband and affected parent) at our laboratory. SpliceAI strongly predicted a new acceptor gain within the inserted sequence (at the second AG in GTAGATC**AG**AAT), with a score of 0.84. This variant has not been reported in any other families with OI. Due to the lack of functional data and the small size of the family, it was initially classified and reported as VUS. Subsequent RT-PCRseq performed on whole blood revealed an in-frame insertion present in the proband (at 18% PSI) and absent in controls (Fig. 4A). (No blood sample was obtained from the affected parent for RNA testing). Notably, all the alternatively spliced cDNA reads began with the AAT sequence from the inserted DNA sequence (GTAGATCAG**AAT**), showing that this partial intron inclusion arose exclusively from the DNA variant. This event falls in the triple helical domain, which is composed of perfect repeats with the sequence glycine-X-Y (G-X-Y), where X and Y are two other amino acids (see Fig. 4B). These repeats form the helical structure that is crucial for the stability of the collagens [22, 23]. Variants that disrupt a glycine residue in *COL1A1* or *COL1A2* are enriched in patients with OI compared to the general population, and glycine-disrupting variants are associated with more severe forms of OI than loss-of-function variants [24]. Therefore, although the PSI in this case is relatively low, the ultimate impact on the protein may have a severe effect on the triple helical domain. Multiple nearby glycine substitutions have been reported in probands with OI, supporting the clinical relevance of this region [25–29]. Combining this evidence with clinical evidence based on the highly specific phenotype of the parent and sibling, we were able to reclassify the variant to Likely Pathogenic [22, 23].

## Discussion

Rare variants predicted to impact splicing, especially those outside a canonical splice dinucleotide, are often classified as VUS due to a paucity of direct clinical or functional evidence [30, 31]. In this work, we present four cases where compelling intronic variants were initially classified as VUS but were upgraded due to RNA analysis performed in our laboratory, providing molecular diagnoses for these individuals. This approach is well suited to rare disease genes, in which many disease-associated variants are underreported or are private to a single family. Because RNA-based functional data can be generated from a single proband or family member and do not rely on clinical or population data, they are applicable for variant classification across a broad range of disease areas, inheritance patterns, and ages of onset or testing. Although the application of evidence codes for RNA studies are not yet standardized, the American College of Medical Genetics recommends that functional evidence can be applied at moderate or strong weighting for studies of this type [32], which has the potential to allow reclassification of VUS suspected to be spliceogenic. In these four cases alone, we were able to provide molecular diagnoses for prenatal, pediatric, and adult-onset cases across a range of skeletal and neurological diseases. Therefore, variant-specific RNA analysis is well poised to complement ES, which is increasingly being used across a variety of indications [33, 34] .

In this case series, we were able to diagnose both probands with a clear family history of disease and those with a more ambiguous clinical history. For the three autosomal dominant conditions, the finding has clear implications for the risk to offspring and other close relatives. In the case with the *COL1A1* variant, we were able to provide a molecular diagnosis for not only the proband but two first-degree relatives with OI. In the autosomal recessive case with the homozygous *ATP6V0A2* variant, we were able to provide accurate recurrence risks for the immediate family; this information may also be valuable for future carrier screening in other relatives.

Although pathogenic splicing variants typically lead to loss of function of the gene product, our work highlights the fact that they can have other mechanisms. For example, the *CSF1R* variant highlighted here impacts a region of the protein that is highly sensitive to in-frame indels involving coding exon 17. Notably, loss of function of *CSF1R* is not a characterized mechanism of disease for the autosomal dominant disorder, which is usually a prerequisite for recommending RNA testing, since the majority of splice defects convey loss of function; however, careful evaluation of the predicted splicing impact of our variant revealed that it was consistent with the impact of many previously reported disease-associated variants, namely disruption of the tyrosine kinase domain via deletion of coding exon 17 with or without accompanying in-frame insertions of novel amino acids [17–21]. Therefore, in certain cases with strong clinical evidence for a particular splicing impact, RNA analysis can be useful for variants in genes where loss of function has not been established as a mechanism of disease.

The *CSF1R* variant also highlights the potential limitations of splice prediction tools and the importance of closely examining variants for potential RNA analysis. The SpliceAI predictions for c.2442+5G>A were negligible, but the position of the variant within the donor site and the clinical evidence for this intronic variant both suggested that it could impact splicing. As shown in Fig. 4, there was a large splicing impact, indicating that SpliceAI, like any prediction tool, occasionally yields false negative predictions.

The use of RNA analysis to re-classify VUS has certain caveats and limitations. First, in many situations, due to availability, blood is a first-tier source for RNA analysis; however, it may not be the most disease-relevant tissue. This limits the genes that can be analyzed to those that are expressed in blood. Also, using RT-PCRseq, it can be difficult to quantitatively detect large events, such as large full- or partial intron retention events. In some cases, variants with strong SpliceAI scores do not show a strong impact on splicing when tested; however, when a predicted splicing event is not observed, it is often difficult to determine whether this is because of the technical limitations of the test (e.g., non-detection of large events) versus a true lack of splice impact. More work testing SpliceAI predictions in higher-throughput studies and studies with multiple modalities is required to understand the scope of this problem. Preliminary data from our laboratory indicate that the majority of spliceogenic VUS that are identified in exome testing and that meet our aforementioned requirements do indeed show altered splicing and can be reclassified [12]. In addition, RNA analysis may lead to identification of a protein effect of uncertain clinical relevance (e.g., a small in-frame deletion of amino acids of unknown function). In this case, the variant may remain VUS even after RNA analysis. Finally, variant-specific RNA analysis requires a longer turnaround time after ES (typically 3–4 weeks at our laboratory), although in our protocol the VUS result is returned while RNA studies are pending, indicating the presence of a candidate cause of disease. Despite these caveats, many cases with compelling VUS can benefit from RNA analysis, as demonstrated herein.

## Conclusions

In all four cases, the candidate variants that were VUS upon initial ES were re-classified to Likely Pathogenic using data from RNA studies. This clearly indicates that variant-specific RNA analysis can add value across a range of indications, inheritance patterns, and even disease mechanisms in rare disease diagnostics, especially as a follow up to ES.

## Methods

### Testing cohort and variant classification

All patients described were evaluated by their health care providers and submitted samples for genetic testing at a single diagnostic laboratory (Ambry Genetics, Aliso Viejo, California). Personal and family history data were collected by patient and/or clinician report from test requisition forms and medical records, where available. A blood draw or buccal swab was performed after obtaining written informed consent for clinical genetic testing. WGC IRB (formerly Western Institutional Review Board) determined the study to be exempt from the Office for Human Research Protections Regulations for the Protection of Human Subjects (45 CFR 46). Clinical ES and variant classification were conducted as previously described [11, 35]. Gene-disease validity scores were determined by an internal team of gene curators using a framework adapted from ClinGen recommendations [11, 36]. SpliceAI [37] scores were used to predict splicing impact, with a score of greater than or equal to 0.1 taken as a non-negligible splice prediction. SpliceAI scores were pre-calculated for the three single nucleotide variants in Cases 1–3 and were calculated at the time of analysis for the delins variant in Case 4.

## RNA analysis

Variants were prioritized for follow-up RNA analysis based on four criteria [12], namely having a SpliceAI score of ≥ 0.2, expression of the relevant gene in whole blood of ≥ 0.5 transcripts per million, an internally curated gene-disease validity of moderate or higher, and a mechanism of disease consistent with the predicted splice impact. Reverse transcriptase polymerase chain reaction sequencing (RT-PCRseq) was performed [38] on RNA from whole blood samples from each proband with custom primers (Table 2) and compared to three same-run, age-matched control samples without a variant in the same gene. Briefly, RNA was extracted from the whole blood PAXgene tube (BD Biosciences, San Jose, CA, USA) samples using a MagMAX RNA Isolation kit (Thermo Fisher Scientific, Chino, CA, USA). The extracted RNA was then purified using a Zymo RNA Clean & Concentrator Kit (Zymo Research, Irvine, CA). One-step reverse transcriptase polymerase chain reaction (RT-PCR) was performed with the SuperScript IV First-Strand Synthesis System (Thermo Fisher Scientific) using 500 ng total RNA and Platinum SuperFi PCR Master Mix (Thermo Fisher Scientific). Cycling included 10 min reverse transcription at 50C and 2 min 98C denaturation, followed by 35 cycles of 10s denaturation at 98C, 10s annealing at 52C, 45s elongation at 72C, and then a final 5 min elongation step at 72C. Amplicons were subject to SPRI purification, quantified on an M200, and 300 ng used for library preparation with KAPA reagents (Kapa Biosystems, Wilmington, MA, USA). Another SPRI purification step preceded library sequencing on an Illumina MiSeq (Illumina, San Diego, CA, USA) with 48 pooled samples. Sequencing reads were aligned to the hg19 reference genome and analyzed using Ambry’s custom bioinformatic pipeline. Percent Splicing Index (PSI) was calculated by the pipeline as described [39]. Due to sample size limitations inherent in this approach, it is not possible to judge the statistical significance of the PSI in probands as compared to controls. The Integrative Genomics Viewer (IGV) [40] was used for visualization of splice junctions and reads. MISO plots were created using a custom modified version of the Sashimi plot utility in the MISO python package [41]. For *COL1A1*, allele-specific expression of the intronic variant was considered as additional evidence of the association of the partial intron retention event with the DNA variant.

**Table 2:** RT-PCRseq primer sequences. Each primer set included a forward or reverse primer that spanned an upstream or downstream exon-exon junction (denoted by an asterisk)

Name	Category	Sequence
ATP6V0A2 R_13.20 s	Forward*	GGCATTGATCCTATTTGGAACT
ATP6V0A2 R_13.20 as	Reverse	CCATAAATCTGAAAGCTTGTTGG
SLC20A2 R_6.11 s2	Forward	TTTGTGTGGCTCTTCGTGTG
SLC20A2 R_6.11 as2	Reverse*	AGCCCACCTTACAGTGCGTG
CSF1R R_14.22 s	Forward	CTCATGTCCGAGCTGAAGAT
CSF1R R_14.22 as	Reverse*	GATTGGTATAGTCCCGCTCTC
COL1A1 R_2.19 s	Forward	GATGACGTGATCTGTGACGAG
COL1A1 R_2.19 as	Reverse*	AGGTTCACCGCTGTTACCC

## Data Availability

The variants analyzed in this study were submitted to ClinVar under the Variation IDs: 1368900, 1334838, 1331662, 447147 and are available at the following URLs: https://www.ncbi.nlm.nih.gov/clinvar/variation/1368900/, https://www.ncbi.nlm.nih.gov/clinvar/variation/1334838/, https://www.ncbi.nlm.nih.gov/clinvar/variation/1331662/, https://www.ncbi.nlm.nih.gov/clinvar/variation/447147/.

## Abbreviations

ES: Exome sequencing
VUS: Variant(s) of uncertain significance
GDV: Gene-disease validity
LOF: Loss of function
RT-PCRseq: RT-PCR sequencing
IGV: Integrative Genomics Viewer
PSI: Percent Splicing Index
MIM: Online Mendelian Inheritance in Man gene identifier
HGNC: HUGO Gene Nomenclature Committee gene identifier
WT: Wildtype
OI: Osteogenesis imperfecta
G-X-Y: Glycine-X-Y
